# Paroxysmal Tachycardia, Epilepsy, Fragilitas Ossium and Mental Retardation

**Published:** 1969-01

**Authors:** J. Jancar

**Affiliations:** Consultant Psychiatrist, Stoke Park Hospital Group, Bristol


### PAROXYSMAL TACHYCARDIA, EPILEPSY, FRAGILITAS OSSIUM, AND MENTAL RETARDATION

BY

J. JANCAR, M.B., B.Ch., B.A.O., D.P.M.

Consultant Psychiatrist, Stoke Park Hospital Group, Bristol

Recently, Derman *et al.* (1968) reported their studies on electroencephalographic abnormalities in patients with paroxysmal tachycardia and their treatment with anti-epileptic drugs. They included in their report similar observations made by other workers (Subbotnik *et al.* 1955, Ringel and Kalistov, 1964 and Pasini *et al.* 1967). Their work shows that patients suffering from paroxysmal tachycardia, without cardiac pathology, have abnormal electroencephalogram recordings, especially in the temporal regions, denoting underlying malfunction in those areas of the brain. They treated this anomaly with sulthiame (' Ospolot ') which is used in the treatment of temporal lobe epilepsy.

I am reporting another unusual case of paroxysmal tachycardia, in which there is no apparent cardiac disease, but there is an association with epilepsy, fragilitas ossium, and severe mental retardation. From the literature available we have, so far, been unable to trace a similar case.

### Case History

A man, 33 years old, sixth child in a family of seven. There is no known mental or physical illness in the family and there was apparently a normal pregnancy and delivery. At his birth his mother's age was 29, and his father's 28. Because of the patient's severe epilepsy, imperfect osteogenesis, and mental retardation he was admitted at the age of 11 years and 9 months to a hospital for the mentally subnormal where he has remained since.

*Mental examination* shows that he has no educational attainments; mental age 3.8 and I.Q. 22. His comprehension and reasoning ability are at the level of between 3 and 4 years. His speech is limited and articulation is very defective.

*Physical examination* reveals signs of fragilitas ossium, blue sclerae, bad posture, and malpositioned limbs due to multiple fractures in the past. He also has bilateral keratoconus. Urine chromatography and blood examinations showed no abnormalities. A skeletal survey shows:—

(a) Spine—numerous old compressions affecting both the dorsal and lumbar vertebrae.

(b) Upper limbs—numerous old and recent fractures in the shafts of the humeri, the left ulna, lower end of right radius and first and fifth metacarpals on the right side.

(c) Hips—marked deformity in the region of the right hip, secondary to an old fracture.

(d) Lower limbs—slight bowing of the right tibia and fibula.

*Electroencephalogram*

(a) Persistent moderately-high amplitude rhythmic 5-6 c/s theta activit from many areas, but most prominent in both fronto-centra regions.

(b) Some moderate-amplitude delta activity from the right occipita region, particularly when the eyes were closed.

(c) The activity in the left temporo-occipital region was much lower i amplitude than from any other region.

Photic stimulation tended to suppress both the theta and the delta activit

*Epilepsy*

The epileptic attacks are of " grand mal " type and are treated wit phenobarbitone and phenytoin.

*Paroxysmal Tachycardia*

The patient's attacks of paroxysmal tachycardia vary in frequency an duration. On average he had one attack every year which lasted from a fe hours to a few days. These had been treated with digoxin, but without succes During one attack an electrocardiogram was recorded which revealed n abnormal function or lesion of the heart except supraventicular tachycard of 220 beats per minute. There is no history or evidence of rheumatic o other cardio-vascular pathology. However, in 1964, he was given 5 ml o paraldehyde intra-muscularly during an attack, and this dramatically broug the pulse rate to a normal level. Since then this treatment has been admin istered as soon as tachycardia has become apparent.

### Summary

The occurrence of paroxysmal tachycardia without a cardiac lesion in a epileptic, mentally severely retarded male patient, also suffering from fragi itas ossium, is reported. Effective treatment with paraldehyde is note Relevant results of observations and investigations are recorded.

Further studies of the relationship between paroxysmal tachycardi abnormal electroencephalogram tracings, and mental retardation are warra ted. So too is a trial of anticonvulsant drugs when there are indications th the cause lies in the nervous system rather than in the heart.

### References

Derman, U., Polvan, Ö., and Sayar, B. (1968) Lancet *ii,* 359.

Pasini, U., Carvalhol, F. E. T., Valada, L., Pileggi, K., Barbato, E.C.D., an Décourt, L. V. (1967) Archos. bras. Cardiol. *20*, 71.

Ringel, J., and Kalistov, I. (1964) Cslká Pediat. *19*, 399.

Subbotnik, S. I., Feniberg, Y. S. and Spielberg, P. I. (1955) Electroence clin. Neurophysiol. *7*, 577.

